# Supplementary material for: A multicenter explanatory survey of patients’ and clinicians’ perceptions of motivational factors in rehabilitation
Source: Commun Med (Lond). 2023 Jun 6;3:78. doi: 10.1038/s43856-023-00308-7 (PMC10244320; doi:10.1038/s43856-023-00308-7)
Supplement: Supplementary file 1 — Description of Additional Supplementary Files [file 43856_2023_308_MOESM1_ESM.pdf]

Description of Additional Supplementary File

File Name: Supplementary Data 1

Description: List of potential motivational factors for clinicians
Note. The potential motivational factors are arranged in alphabetical order.

File Name: Supplementary Data 2

Description: The survey questions presented to clinicians

File Name: Supplementary Data 3

Description: Characteristics of participants
Note. Values are presented as the number (%) or median [interquartile range].

File Name: Supplementary Data 4

Description: Associations between patients' choices regarding the most important motivational factor and their demographic characteristics
Note. The potential motivational factors are arranged in descending order by the number of participants who selected each factor. The N column denotes the number of participants who selected the relevant item. Values are presented as the odds ratio (95% confidence interval). The odds ratio values marked in bold show a significant association between patients' choice and demographic characteristics. Not estimable indicates that the odds ratio was not estimated because there were no patients who corresponded to the reference group.
* Reference group of patients with fracture.
† Reference group of female patients.
‡ Reference group of patients < 65 years of age.

File Name: Supplementary Data 5

Description: Distributions of patients' and clinicians' answers regarding the most important motivational factor
Note. The potential motivational factors are arranged in descending order by the percentage of participants who selected each factor as the most important.

File Name: Supplementary Data 6

Description: Distribution of patients' and clinicians' answers regarding the three most important motivational factors
Note. The potential motivational factors are arranged in descending order by the percentage of participants who selected each factor as the most important.
