# Supplementary material for: A multicenter explanatory survey of patients’ and clinicians’ perceptions of motivational factors in rehabilitation
Source: Commun Med (Lond). 2023 Jun 6;3:78. doi: 10.1038/s43856-023-00308-7 (PMC10244320; doi:10.1038/s43856-023-00308-7)
Supplement: Supplementary file 2 — Supplementary information [file 43856_2023_308_MOESM2_ESM.pdf]

Supplementary material

A Multicenter Explanatory Survey of Patients' and Clinicians' Perceptions of Motivational Factors in Rehabilitation

Authors:

Kazuaki Oyake,[1,2] Katsuya Yamauchi,[3] Seigo Inoue,[2] Keita Sue,[4] Hironobu Ota,[5] Junichi Ikuta,[6] Toshiki Ema,[7] Tomohiko Ochiai,[8] Makoto Hasui,[9] Yuya Hirata,[10] Ayaka Hida,[11] Kenta Yamamoto,[12] Yoshihiro Kawai,[13] Kiyoto Shiba,[14] Akihito Atsumi,[15] Tetsuyuki Nagafusa,[3] Satoshi Tanaka[16]

Affiliations:

[1] Department of Physical Therapy, School of Health Sciences, Shinshu University, Nagano, Japan

[2] Department of Rehabilitation Medicine, Tokyo Bay Rehabilitation Hospital, Chiba, Japan

[3] Department of Rehabilitation Medicine, Hamamatsu University School of Medicine, Shizuoka, Japan

[4] Department of Rehabilitation, Kakeyu-Misayama Rehabilitation Center Kakeyu Hospital, Nagano, Japan

[5] Rehabilitation Center, Aichi Medical University Medical Center, Aichi, Japan

[6] Division of Occupational Therapy, Department of Rehabilitation, Nakaizu Rehabilitation Center, Shizuoka, Japan

[7] Department of Physical Therapy, Suzukake Central Hospital, Shizuoka, Japan

[8] Rehabilitation Center, Juzen Memorial Hospital, Shizuoka, Japan

[9] Department of Rehabilitation Medicine, JA Shizuoka Kohseiren Enshu Hospital, Shizuoka, Japan

[10] Department of Rehabilitation, Suzukake Healthcare Hospital, Shizuoka, Japan

[11] Department of Rehabilitation Medicine, Kakegawa Higashi Hospital, Shizuoka, Japan

[12] Department of Rehabilitation, Toyoda Eisei Hospital, Shizuoka, Japan

[13] Department of Rehabilitation, Tenryu Suzukake Hospital, Shizuoka, Japan

[14] Department of Rehabilitation Medicine, Hamakita Sakuradai Hospital, Shizuoka, Japan

[15] Department of Rehabilitation, Hamamatsu-Kita Hospital, Shizuoka, Japan

[16] Laboratory of Psychology, Hamamatsu University School of Medicine, Shizuoka, Japan

Table S1. List of potential motivational factors for patients

Potential motivational factors
Active listening
A suitable rehabilitation environment
Control of task difficulty
Enjoyable rehabilitation programs
Feedback regarding the results of the practice
Goal setting
Group rehabilitation
Medical information
Practice related to the patient’s experience and lifestyle
Praise
Presence of family members during rehabilitation
Realization of recovery
Rehabilitation programs with variations
Respect for self-determination
Rewards for effort
Other

Table S2. Structured interview guide

**First question**<br>From the list, please select the three items that you consider to be most important for facilitating your engagement in rehabilitation. If you have another most important factor to increase your motivation for rehabilitation besides the 15 items on the list, please select “other.”
**Second question**<br>Among your three selections, which is your top choice?
**If the patient selects “other” in the first or second question, the interviewer asks the patient a third question.**
**Third question**<br>Please tell me an additional motivational factor that corresponds to “other.”

Table S3. Additional factors motivating patients to engage in rehabilitation proposed by patients

Motivational factors	N
A good relationship with therapists	2
Clinician’s efforts to help me	2
Encouragement from therapists	1
Explanation of my expected length of hospitalization	1
Good blood pressure in the morning	1
Improvement in my abilities	1
My willingness	1
Nothing in particular	2
Pleasant massage	1

The reported motivational factors are arranged in alphabetical order.

The N column denotes the number of participants.

a The distribution of patients' answer (n = 240)

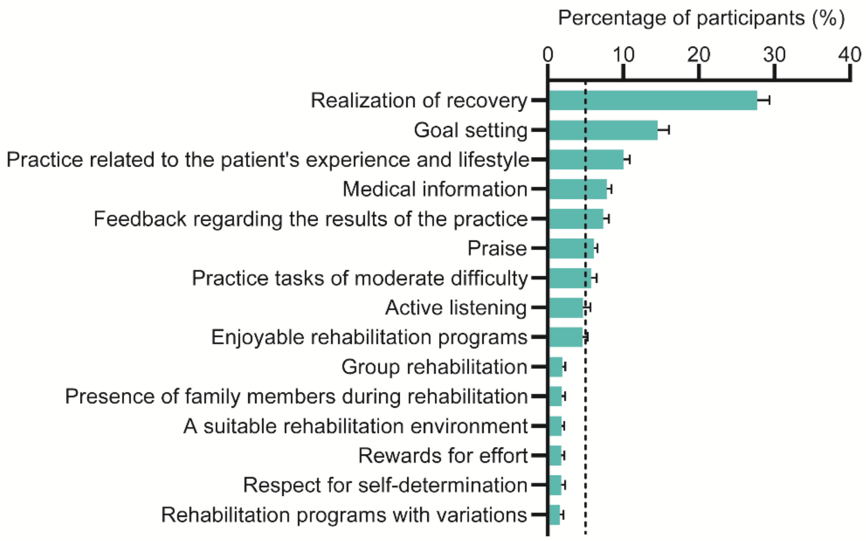

b The distribution of clinicians' answer (n = 200)

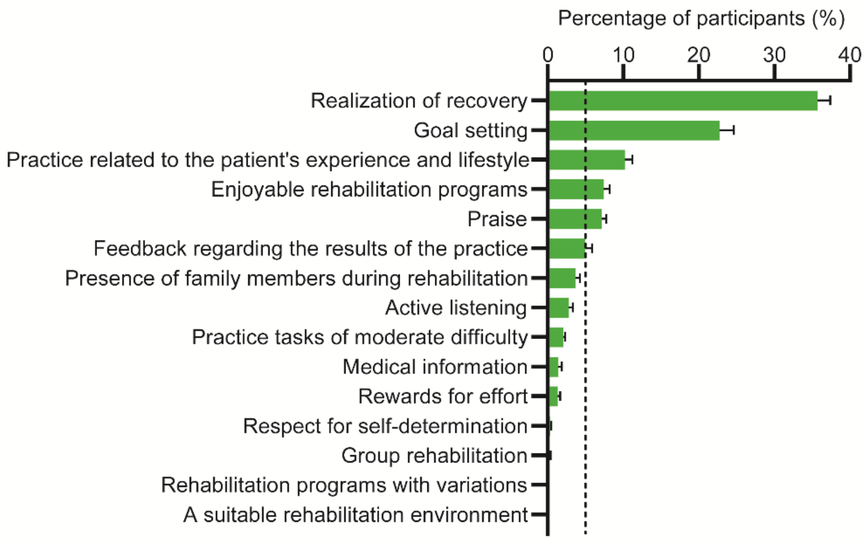

Figure S1. Distributions of patients' (a) and clinicians' (b) answers regarding the most important motivational factor obtained by randomly sampling approximately half of the participants (240 patients and 200 clinicians) 10 times with replacement from all participants. The potential motivational factors are arranged in descending order by the mean of the percentage of participants. Error bars represent 95% confidence intervals. The vertical dashed line represents 5% of participants.

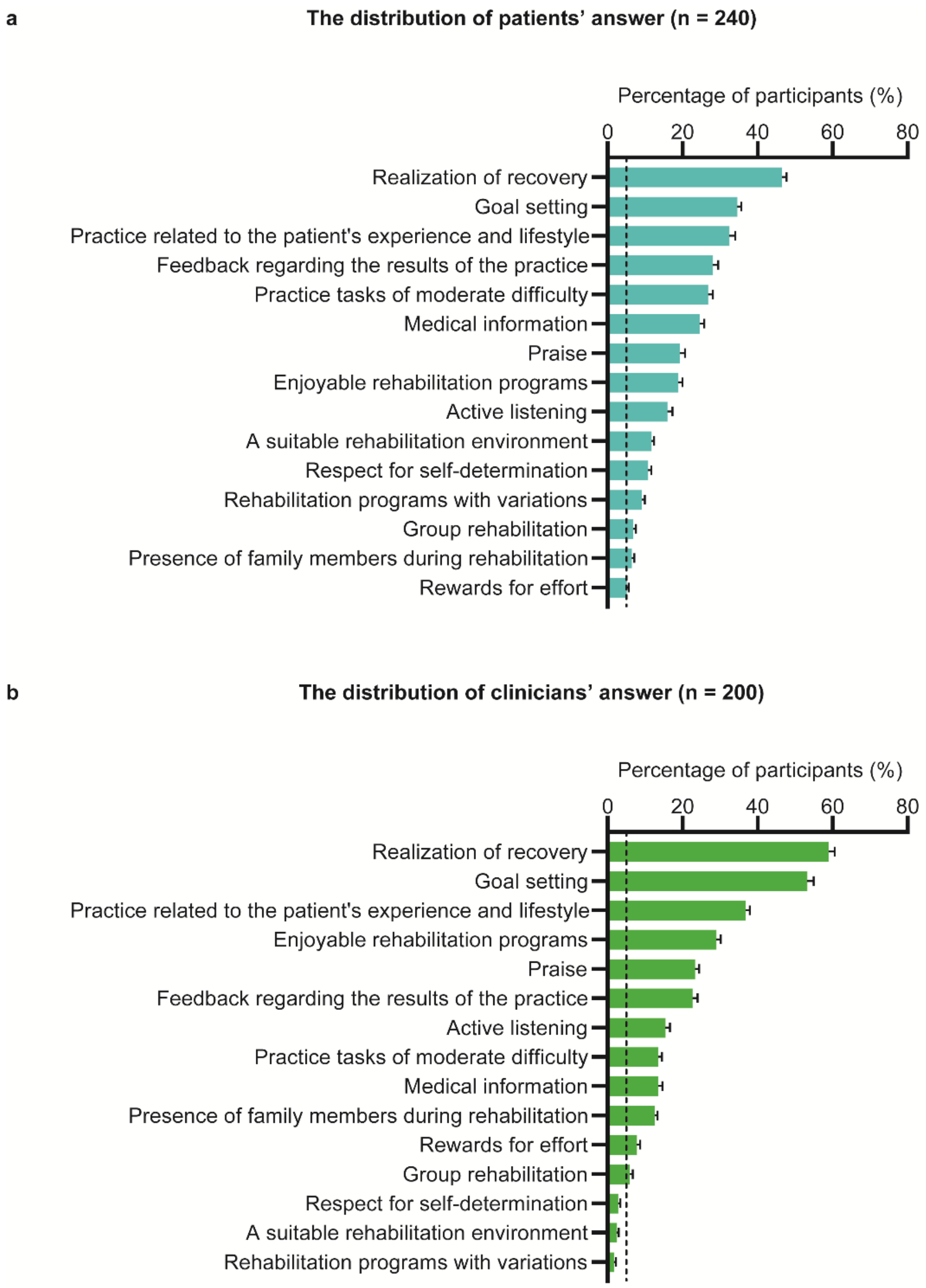

Figure S2. Distribution of patients' (a) and clinicians' (b) answers regarding the three most important motivational factors obtained by repeated random sampling with replacement from all participants.

The potential motivational factors are arranged in descending order by the mean of the percentage of participants. Error bars represent 95% confidence intervals. The vertical dashed line represents 5% of participants.
