# Supplementary material for: A multicenter explanatory survey of patients’ and clinicians’ perceptions of motivational factors in rehabilitation
Source: Commun Med (Lond). 2023 Jun 6;3:78. doi: 10.1038/s43856-023-00308-7 (PMC10244320; doi:10.1038/s43856-023-00308-7)
Supplement: Supplementary file 10 — Reporting Summary [file 43856_2023_308_MOESM10_ESM.pdf]

nature portfolio

Corresponding author(s): DBPR and COMMSMED-22-0419B

Last updated by author(s): May 15, 2023

Reporting Summary

Nature Portfolio wishes to improve the reproducibility of the work that we publish. This form provides structure for consistency and transparency in reporting. For further information on Nature Portfolio policies, see our Editorial Policies and the Editorial Policy Checklist.

Statistics

For all statistical analyses, confirm that the following items are present in the figure legend, table legend, main text, or Methods section.

n/a	Confirmed	
☐	☒	The exact sample size (n) for each experimental group/condition, given as a discrete number and unit of measurement
☐	☒	A statement on whether measurements were taken from distinct samples or whether the same sample was measured repeatedly
☐	☒	The statistical test(s) used AND whether they are one- or two-sided<br>*Only common tests should be described solely by name; describe more complex techniques in the Methods section.*
☐	☒	A description of all covariates tested
☐	☒	A description of any assumptions or corrections, such as tests of normality and adjustment for multiple comparisons
☐	☒	A full description of the statistical parameters including central tendency (e.g. means) or other basic estimates (e.g. regression coefficient) AND variation (e.g. standard deviation) or associated estimates of uncertainty (e.g. confidence intervals)
☐	☒	For null hypothesis testing, the test statistic (e.g. F, t, r) with confidence intervals, effect sizes, degrees of freedom and P value noted<br>*Give P values as exact values whenever suitable.*
☒	☐	For Bayesian analysis, information on the choice of priors and Markov chain Monte Carlo settings
☒	☐	For hierarchical and complex designs, identification of the appropriate level for tests and full reporting of outcomes
☒	☐	Estimates of effect sizes (e.g. Cohen's d, Pearson's r), indicating how they were calculated

Our web collection on statistics for biologists contains articles on many of the points above.

Software and code

Policy information about availability of computer code

Data collection	Google Forms tool, Google LLC, Mountain View, CA, USA
Data analysis	Statistical Package for the Social Sciences software version 27.0, International Business Machines Corp., NY, USA

For manuscripts utilizing custom algorithms or software that are central to the research but not yet described in published literature, software must be made available to editors and reviewers. We strongly encourage code deposition in a community repository (e.g. GitHub). See the Nature Portfolio guidelines for submitting code & software for further information.

Data

Policy information about availability of data

All manuscripts must include a data availability statement. This statement should provide the following information, where applicable:

- Accession codes, unique identifiers, or web links for publicly available datasets
- A description of any restrictions on data availability
- For clinical datasets or third party data, please ensure that the statement adheres to our policy

The datasets used and/or analyzed during the current study are available from the corresponding author on reasonable request because of participant confidentiality. The numerical data underlying the Figures 1 and 3 are shown in Supplementary Data 5 and 6, respectively.

Human research participants

Policy information about studies involving human research participants and Sex and Gender in Research.

Reporting on sex and gender	The findings of the study can apply to both male and female. Sex was not considered in study design. In patient participants, demographic data including sex was obtained from medical records. Sex of clinician participants was determined based on self-reporting. We examined the association between patients' choices regarding the most important motivational factor with their demographic characteristics including sex.
Population characteristics	See above.
Recruitment	From 13 hospitals with an intensive inpatient rehabilitation ward, participants were selected purposively based on the inclusion criteria. No self-selection or other biases are known or expected.
Ethics oversight	This study protocol was approved by the appropriate ethics committee at the Hamamatsu University School of Medicine (approval number: 21-233).

Note that full information on the approval of the study protocol must also be provided in the manuscript.

Field-specific reporting

Please select the one below that is the best fit for your research. If you are not sure, read the appropriate sections before making your selection.

☐ Life sciences ☒ Behavioural & social sciences ☐ Ecological, evolutionary & environmental sciences

For a reference copy of the document with all sections, see nature.com/documents/nr-reporting-summary-flat.pdf

Behavioural & social sciences study design

All studies must disclose on these points even when the disclosure is negative.

Study description	A multicenter explanatory survey research design.
Research sample	Patients hospitalized in an intensive inpatient rehabilitation ward were recruited through purposive sampling based on the inclusion criteria from 12 hospitals in Japan. Inclusion criteria were as follows: being aged 20 to 90 years, having an established diagnosis of neurological or orthopedic disorders as the primary reason for hospitalization, having undergone an inpatient rehabilitation program for at least four weeks at the time of study participation, and adequate communication skills to complete the questionnaire.<br>Clinicians were also purposively sampled from 13 hospitals in Japan. They included physicians, physical therapists, occupational therapists, or speech-language-hearing therapists working in an intensive inpatient rehabilitation ward. Patients were recruited as participants from 12 of these hospitals.<br>The primary reason for hospitalization of most patients was either stroke (45.5%) or fracture (42.2%). Approximately half of the clinicians were physical therapists (49.9%).<br>All of the participants were recruited in Japan, potentially limiting the international generalizability of our findings. Nevertheless, the present results support the results of previous studies from different countries. The opinions of patients with stroke and patients with fracture might have been overstated in the current sample. Similarly, the responses of physical therapists may have been overstated in clinicians' perceptions. However, the percentage of patients with stroke, patients with fracture, and physical therapists in our sample may be consistent with the actual situation.
Sampling strategy	Participants were selected selected purposively based on the inclusion criteria. The sample size calculation for participants was based on epidemiological data from the Kaifukuki Rehabilitation Ward Association. Totally, 38,363 patients with neurological and orthopedic diseases were admitted to intensive inpatient rehabilitation wards. Of them, 18,870 patients had neurological diseases and 19,493 had orthopedic diseases. Similarly, the total number of rehabilitation clinicians working in the intensive inpatient rehabilitation wards was estimated at 66,033, with 30,911 physical therapists, 18,700 occupational therapists, 8,843 physicians, and 7,579 speech-language-hearing therapists. Based on these population sizes and using a margin of error of 5% at a 95% confidence interval, the estimated minimum sample size was 381 patients and 382 clinicians for this study.
Data collection	We administered the patient questionnaire in an interview style, and patients took part in a face-to-face structured interview with a researcher at each hospital. The patient survey was available in paper form. In each hospital, one researcher was responsible for administering the survey. Following the end of the recruitment period, all completed questionnaires were mailed to the first author.<br>We used a cloud-based questionnaire and survey software (Google Forms tool, Google LLC, Mountain View, CA, USA) to develop the clinician survey and to collect data. To publicize the study, the researcher at each hospital distributed leaflets to clinicians who met the inclusion criteria. The leaflets contained a brief description of the study and a hyperlink to the survey. Clinicians could voluntarily access the survey website using their own laptops, tablets, or smartphones.<br>The researcher was not blind to experimental condition and the study hypothesis during data collection.
Timing	The survey was conducted from January to March 2022.

Data exclusions	No data were excluded from the analyses.
Non-participation	Of the total of 520 patients who met the inclusion criteria, 23 refused to participate in this study. In addition, of the 645 clinicians who met the inclusion criteria, 401 responded. The response rates of the patient and clinician surveys were 92.1% and 62.2%, respectively. No participants dropped out/declined participation.
Randomization	Participants were not allocated into experimental groups.

Reporting for specific materials, systems and methods

We require information from authors about some types of materials, experimental systems and methods used in many studies. Here, indicate whether each material, system or method listed is relevant to your study. If you are not sure if a list item applies to your research, read the appropriate section before selecting a response.

Materials & experimental systems

n/a	Involved in the study
☒	☐ Antibodies
☒	☐ Eukaryotic cell lines
☒	☐ Palaeontology and archaeology
☒	☐ Animals and other organisms
☒	☐ Clinical data
☒	☐ Dual use research of concern

Methods

n/a	Involved in the study
☒	☐ ChIP-seq
☒	☐ Flow cytometry
☒	☐ MRI-based neuroimaging
